# Diagnostic Agreement between Prehospital Emergency and In-Hospital Physicians

**DOI:** 10.1155/2019/3769826

**Published:** 2019-04-24

**Authors:** Nikolai Ramadanov, Roman Klein, Fabian Laue, Wilhelm Behringer

**Affiliations:** ^1^Center for Emergency Medicine, University Hospital Jena, Friedrich Schiller University Am Klinikum 1, 07747 Jena, Germany; ^2^Clinic for Reconstruction and Trauma Surgery, Ernst von Bergmann Hospital Charlottenstr. 72, 14467 Potsdam, Germany; ^3^Orthopaedics, Trauma Surgery and Sports Traumatology, Marienhausklinikum Hetzelstift, Stiftstr. 10, 67434 Neustadt, Germany

## Abstract

**Background:**

The aim of the study was to determine the diagnostic agreement between the discharge diagnosis and the suspected diagnosis by the prehospital emergency physician and to run a sensitivity analysis of the most common diagnoses by the prehospital emergency physician.

**Methods:**

The diagnostic agreement was determined by a systematic comparison of the discharge diagnosis with suspected diagnosis by the prehospital emergency physician in a period of 24 months at the emergency medical services in Bad Belzig. The diagnostic agreement of the 13 most common discharge diagnoses was compared to the remaining diagnostic agreement. The results were tested for statistical significance using the chi-squared test.

**Results:**

In 64.1% of cases included, a diagnostic agreement occurred. There was a high proportion of diagnostic agreement for hypoglycemia (97%), atrial fibrillation (87%), cramping seizure (86%), hypertensive crisis (85.5%), and syncope (81%). There was a low proportion of diagnostic agreement for chest wall pain (27%), pneumonia (32%), and cardiac decompensation (53%).

**Conclusions:**

Our attention in practice and emergency medical courses should be directed to chest pain patients and the main symptom of dyspnea, because of the high proportion of incorrect diagnoses by the prehospital emergency physician. It should be noted that 92% of incorrectly diagnosed chest wall pain cases were overestimated with an acute coronary syndrome.

## 1. Introduction

Due to the lack of laboratory-chemical examinations and imaging diagnostics in the emergency medical services (EMS), the correct prehospital diagnosis is a challenge for the prehospital emergency physician. Nevertheless, due to the often life-saving nature of prehospital missions, the correct diagnosis is important to the patient outcome. There are several studies done about the diagnostic agreement (dA) in the EMS and in the emergency department (ED) [1–4]. The results for dA showed a very broad spectrum (46.5%-90%). It should be noted that in these studies the methods for determining dA were different. A breakdown and sensitivity analysis of the most common diagnoses by the prehospital emergency physician has not yet been performed. It is of utmost importance for emergency medical science to know which clinical pictures have a low diagnostic agreement in prehospital missions.

The aim of our study was to determine the dA between emergency mission-related discharge diagnoses and suspected diagnoses by the prehospital emergency physician and to perform a sensitivity analysis of the most common emergency mission-related discharge diagnoses.

## 2. Study Design and Examination Methods

### 2.1. Data Collection

All prehospital emergency physician's patient care reports (DIVI protocol 4.2) of the EMS in Bad Belzig and the corresponding discharge summaries from the hospital information system in Bad Belzig (SAP Version 7400.1.0.1093 and Cerner Soarian Clinicals Version 4.1) in the period from July 1, 2013, to June 30, 2014, and from January 1, 2015, to December 31, 2015, were examined. Further discharge summaries were included from neighboring hospitals (Klinikum Ernst von Bergmann Potsdam, Asklepios Fachklinik Brandenburg, Städtisches Krankenhaus Brandenburg, Johanniter Krankenhaus in Fläming Treuenbrietzen). The study was approved by the ethical committee of the University of Jena (No. 4522-08/15/15).

### 2.2. Diagnostic Agreement

First, all prehospital mission-related discharge diagnoses from the 1055 included cases were determined. In case that the discharge diagnosis from the discharge summaries for the corresponding mission could be confirmed in the suspected diagnoses from the patient care reports by the prehospital emergency physician, a dA occurred. In case that the discharge diagnosis from the discharge summaries for the corresponding mission could not be confirmed in the suspected diagnosis from the patient care reports by the prehospital emergency physician, a dA did not occur. This systematic comparison established the dA for all 1378 prehospital mission-related discharge diagnoses. It was done by two experienced emergency physicians (N. Ramadanov and F. Laue) independently from each other using the ICD 10 coding (*κ* = 0,95). In divergent cases a third emergency physician (W. Behringer) helped find the correct adjudication.

### 2.3. Statistics

As part of a sensitivity analysis, the dA of the 13 most common prehospital mission-related hospital discharge diagnoses was tested for statistically significant differences compared to the remaining dA. The chi-square test was used with a significance level of p = 0.05. Statistical calculations were performed using IBM SPSS Statistics 19 for Windows.

## 3. Results

### 3.1. Exclusion of Cases

**Figure 1:**
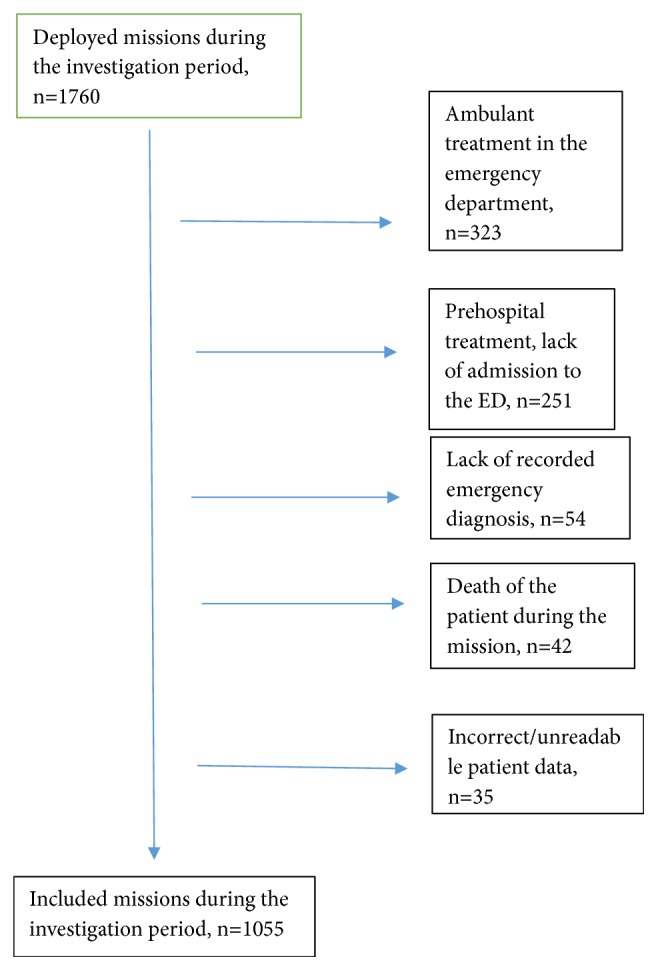
Inclusion chart.

705 patient care reports were excluded from the study for the following reasons: ambulant treatment in the emergency department; prehospital treatment, lack of admission to the ED; lack of recorded emergency diagnosis; death of the patient during the mission or incorrect/unreadable patient data (see Figure 1).

### 3.2. Patients and Prehospital Emergency Physicians

The average age of the 1055 patients was 70 years (min. 1, max 100 years). 493 of them were male and 562 were female. 64.5% of the prehospital missions were carried out by internist prehospital emergency physicians, 27% by surgical, 7% by general practitioners, and 1.5% by anesthetic emergency physicians. 70% of the prehospital missions were carried out by resident physicians and 30% by specialist physicians. 33% of the prehospital missions were carried out by prehospital emergency physicians with a higher approval for emergency medicine (“Zusatzbezeichnung Notfallmedizin”) and 67% of the prehospital missions by prehospital emergency physicians with a lower approval for emergency medicine (“Fachkunde Rettungsdienst”).

### 3.3. Spectrum of Discharge Diagnoses

**Figure 2:**
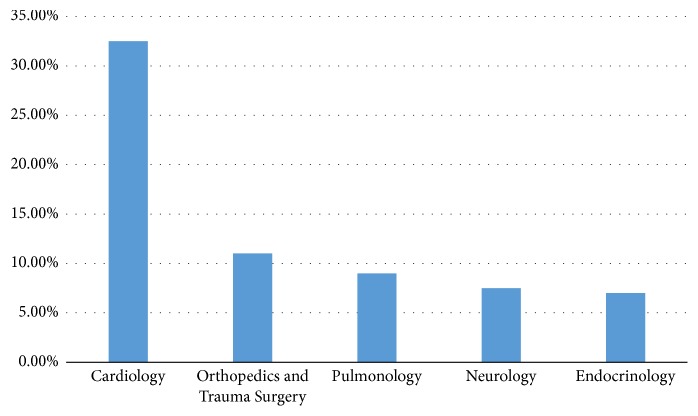
Distribution of the prehospital mission-related hospital discharge diagnoses by the prehospital emergency physician, summarized by specialty.

At 1055 included cases, a total of 1378 prehospital mission-related hospital discharge diagnoses were formulated after emergency medical admission. In 238 cases there were made 2 discharge diagnoses for one patient, in 38 cases 3 discharge diagnoses and in 3 cases 4 discharge diagnoses for one patient.  Table 1 shows the 13 most common prehospital mission-related discharge diagnoses after emergency medical admission and Figure 2 the distribution of discharge diagnoses by specialty.

### 3.4. Determined Diagnostic Agreement and Sensitivity Analysis

A dA occurred in 64.1% and did not occur in 35.9% of cases. The results for the sensitivity analysis of the 13 most common prehospital mission-related hospital discharge diagnoses are listed in Table 2.

### 3.5. Accumulation of dA

An accumulation of dA among the 13 most common prehospital mission-related hospital discharge diagnoses was determined for hypoglycemia with 97% (Chi-square = 16.69; DF = 2; p = 0,01), arrhythmia absoluta with 87% (Chi-square = 9.36; DF = 1; p = 0,01), seizure with 86% (Chi-square = 7.84; DF = 1; p = 0,01), hypertensive crisis with 85,5% (Chi-square = 25,57; DF = 1; p = 0,01), and syncope with 81% (Chi-square = 11,15; DF = 1; p = 0,01).

### 3.6. Lack of dA

Table 4 showed a low proportion of dA for chest wall pain with 27% compared to the dA of the rest of diagnoses (chi-square = 19.8, DF = 1, p = 0.01). In 92% of cases of missing dA chest wall pain was considered incorrectly as acute coronary syndrome.  Table 5 showed a low proportion of dA for pneumonia with 68% compared to the dA of the rest of diagnoses (chi-square = 24.34, DF = 1, p = 0.01). In 30% of cases of missing dA, pneumonia was considered incorrectly as pulmonary edema and cardiac decompensation.  Table 6 showed a low proportion of dA for cardiac decompensation with 47% compared to the dA of the rest of diagnoses (chi-square = 5.12, DF = 1, p = 0.02). In 22% of cases of missing dA, cardiac decompensation was considered incorrectly as exacerbated COPD.

## 4. Discussion

The most common prehospital mission-related hospital discharge diagnoses were made in cardiopulmonary diseases (42%), followed by the specialties orthopedics and trauma surgery (11%), neurology (8%), and endocrinology (7.5%). The most common prehospital mission-related hospital discharge diagnoses were hypertensive crisis (8.5%), cardiac decompensation (6.8%), syncope (6.1%), dehydration (5.2%), and acute coronary syndrome (4.1%).

Calculating the dA, it was possible to estimate retrospectively the correctness of the suspected diagnosis by the prehospital emergency physician. Based on 1055 included missions, our findings showed that the suspected diagnosis was correct in 64.1% of the cases. The sensitivity analysis of the 13 most common prehospital mission-related hospital discharge diagnoses showed a significantly high proportion of correct diagnoses for hypoglycemia (97%), arrhythmia absoluta (87%), seizure (86%), hypertensive crisis (85.5%), and syncope (81%) and a significantly high proportion of incorrect diagnoses for chest wall pain (73%), pneumonia (68%), and cardiac decompensation (47%).

### 4.1. Accumulation of Correct Suspected Diagnoses by the Prehospital Emergency Physician

For diseases with a very remarkable clinical picture such as seizure and syncope, the accumulation of correct suspected diagnosis is understandable. The accumulation of correct suspected diagnoses by the prehospital emergency physician for hypoglycemia, hypertensive crisis, and arrhythmia absoluta can be explained by the fact that there is preclinical possibility for precise diagnostics (blood glucose measurement, blood pressure measurement, 12-channel ECG) in these diseases. The impact of the lack of laboratory and imaging diagnostics in prehospital medicine can be seen in the comparison of our present results with the study by Dormann with similar methods for the determination of the dA. Among other things, the admission diagnosis from the emergency department was compared to the hospital discharge diagnosis in his study [3]. In 4321 hospitalized patients, the dA was 71%, well above the dA of the present study (64.1%), which can be explained simply by the lack of opportunities for laboratory-chemical examinations and imaging diagnostics.

### 4.2. Lack of Correct Suspected Diagnoses by the Prehospital Emergency Physician for Chest Wall Pain

The chest wall pain represents a prehospital diagnostic challenge with a low proportion of 27% of correct suspected diagnoses by the prehospital emergency physician. This was already discovered in numerous other studies [7, 8]. Our findings showed that in 92% of the cases of chest wall pain the prehospital suspected diagnoses by the prehospital emergency physician were considered as an acute coronary syndrome and were accordingly mistreated with drugs (Heparin, acetylsalicylic acid). “Most patients with “pectanginal” symptoms do not suffer from a life-threatening heart disease” [9]. In a study with 1212 “chest pain patients”, who visited a GP practice, only 3.6% had an acute coronary syndrome [10]. Obviously the prehospital chest pain needs more attention being a prehospital differential diagnostic challenge. A more accurate medical history and physical examination should improve the correctness of the suspected diagnosis.  Table 3 is reproduced from McConaghy et al. (2013) [under the Creative Commons Attribution License/https://www.aafp.org/afp/2013/0201/p177.html]. It shows a possible differential diagnostic procedure for chest pain patients that was created after evaluation of 13 other studies [5, 6, 8, 11–14].

### 4.3. Lack of Correct Diagnoses for Main Symptom “Dyspnea”

Pneumonia with 32% and cardiac decompensation with 53% showed a low proportion of correct suspected diagnoses by the prehospital emergency physician. Furthermore a high proportion of diseases from the cardiopulmonary area with common main symptom “dyspnea” was noticed during the evaluation of the prehospital mission-related hospital discharge diagnoses. So the main symptom “dyspnea” presents a prehospital diagnostic challenge because of its diverse causes. This was already determined in other studies [10, 15].

### 4.4. Limitations

There is a certain degree of subjectivity in the determination of dA in this study as well as in other studies, since the choice of different methods may lead to deviations in the results. Since there are differences in the EMS system over the whole country, the described EMS location is not sufficiently representative. A geographically varying distribution of the diagnoses is conceivable. Transferring the results to other EMS locations and regions is therefore difficult.

## 5. Conclusion

(i) Special attention in the prehospital mission and during the education of prehospital emergency physicians should be paid to chest pain patients due to the accumulation of misdiagnosis, as 92% of incorrectly diagnosed patients with chest wall pain were overestimated and mistreated as cases with acute coronary syndrome.

(ii) Special attention in the prehospital mission and during the education of prehospital emergency physicians should be paid as well on cardiopulmonary diseases with the common main symptom “dyspnea” as a diagnostic challenge.

## Figures and Tables

**Table 1:** The 13 most common prehospital mission-related hospital discharge diagnoses by the prehospital emergency physician (n = 1378).

ICD	Designation	%	Number
I10.91	Hypertensive crisis	8.5	117
I50.9	Cardiac decompensation	6.8	94
R55, I95.9	Syncope, hypotonic dysregulation	6.1	84
E86	Dehydration	5.2	71
I21.9	Acute coronary syndrome	4.1	57
J15.9	Pneumonia	3.8	53
J44.09	Exacerbated COPD	3.4	47
I48.9	Arrhythmia absoluta	2.8	39
I20.0	Stable angina pectoris	2.6	36
G40.9, R56.8, R56.0	Seizure	2.6	36
R07.4, G58.8	Chest wall pain	2.4	33
E16.2/E15	Hypoglycemia/hypoglycemic coma	2.2	31
I63.9	Stroke	1.9	26

**Table 2:** Sensitivity analysis of the most common prehospital mission-related hospital discharge diagnoses by the prehospital emergency physician.

Designation	Number of dA (n)		p
	Yes	No	
Diagnoses total	64,1% (883)	35,9% (495)	-
Hypertensive crisis	85,5% (100)	14,5% (17)	0,01
Cardiac decompensation	53% (50)	47% (44)	0,02
Syncope, hypotonic dysregulation	81% (68)	19% (16)	0,01
Dehydration	-	-	0,89
Acute coronary syndrome	-	-	0,88
Pneumonia	32% (17)	68% (36)	0,01
Exacerbated COPD	-	-	0,12
Arrhythmia absoluta	87% (34)	13% (5)	0,01
Stable angina pectoris	81% (29)	19% (7)	0,04
Seizure	86% (31)	14% (5)	0,01
Chest wall pain	27% (9)	73% (24)	0,01
Hypoglycemia/hypoglycemic Coma	97% (30)	3% (1)	0,01
Stroke	-	-	0,57

**Table 3:** Differential diagnosis of chest pain, adapted from Conaghy et al.

Acute myocardial infarction	Chest pain radiates to both arms	7.1	0.67
	Third heart sound on auscultation	3.2	0.88

	Hypotension	3.1	0.96

Chest wall pain	At least two of the following findings: localized muscle tension; stinging pain; pain reproducible by palpation; absence of cough	3.0	0.47

Gastroesophageal reflux disease	Burning retrosternal pain, acid regurgitation, sour or bitter taste in the mouth; one-week trial of high-dose proton pump inhibitors relieves symptoms	3.1	0.30

Panic disorder/anxiety state	Single question: in the past four weeks, have you had an anxiety attack (suddenly feeling fear or panic)?	4.2	0.09

Acute thoracic aortic dissection	Acute chest or back pain and a pulse differential in the upper extremities	5.3	NA

Note: The higher the LR is above 1, the better it rules in disease (greater than 10 is considered good). Conversely, the lower the LR is below 1, the better it rules out disease (less than 0.1 is considered good). LR+ = positive likelihood ration; LR- = negative likelihood ratio; NA = not available. Information is from references [5] through [6].

**Table 4:** dA for “chest wall pain” compared to the remaining diagnoses.

	Number of dA (n)		p
	Yes	No	
Chest wall pain	27% (9)	73% (24)	0,01
Rest	65% (874)	35% (468)	

**Table 5:** dA for “pneumonia” compared to the remaining diagnoses.

	Number of dA (n)		p
	Yes	No	
Pneumonia	32% (17)	68% (36)	0,01
Rest	65% (866)	35% (459)	

**Table 6:** dA for “cardiac decompensation” compared to the remaining diagnoses

	Number of dA (n)		p
	Yes	No	
Cardiac decompensation	53% (50)	47% (44)	0,02
Rest	65% (833)	35% (451)	

## Data Availability

The datasets used and/or analysed during the current study are available from the corresponding author upon reasonable request.

## Abbreviations

dA: Diagnostic agreement
COPD: Chronic obstructive pulmonary disease
DIVI: Deutsche interdisziplinäre Vereinigung für Intensiv- und Notfallmedizin
EMS: Emergency medical services
ED: Emergency department
GP: General practitioner
IBM SPSS: International Business Machines Corporation Superior Performing Software System
SAP: Systeme, Anwendungen, Produkte.
